# Rural-Urban Differences in the Social Determinants of Health Among Custodial Grandparents, 2018-2023

**DOI:** 10.1093/geroni/igaf122.887

**Published:** 2025-12-31

**Authors:** Tenesha Littleton, Orion Mowbray, Joana Okine

**Affiliations:** The University of Alabama, Tuscaloosa, Alabama, United States; University of Georgia, Athens, Georgia, United States; The University of Alabama, Tuscaloosa, Alabama, United States

## Abstract

Custodial grandparents report worse mental and physical health than their non-caregiving counterparts. Prior research indicates that custodial grandparents’ health and well-being are adversely impacted by the social determinants of health (SDOH), including economic hardship and social isolation. However, prior research on the SDOH among custodial grandparents is limited by small sample sizes and a lack of focus on geographic variations in these experiences. Compared to custodial grandparents in urban settings, rural dwelling custodial grandparents may face additional challenges that impede health due to remote location and lack of social services within rural settings. The purpose of this study was to examine rural-urban differences across multiple SDOH indicators (social support, economic hardship, neighborhood built and social environment) and determine if these indicators were stable across time. Data on custodial grandparents were obtained from the 2018-2023 National Survey of Children’s Health (n = 11,355). About 16% of the sample lived in rural areas. The average age was 60 and most of the sample was female (79%). Controlling for demographics, a multivariate logistic regression model examined the association between rural/urban residence and SDOH indicators. Custodial grandparents in rural areas showed lower odds of better neighborhood-built environment (OR = 0.75) and higher odds of better neighborhood social environments (OR = 1.11). From 2018 to 2023, changes in built and social environment scores among custodial grandparents in both rural and urban areas were not significant. Improving the built environment for rural custodial grandparents is crucial to enhancing well-being and guiding caregiver-focused interventions, policies, and programs.

